# Positive cfDNA screening results for 22q11.2 deletion syndrome—Clinical and laboratory considerations

**DOI:** 10.3389/fgene.2023.1146669

**Published:** 2023-03-10

**Authors:** Erica Soster, Brittany Dyr, Jill Rafalko, Eyad Almasri, Phillip Cacheris

**Affiliations:** ^1^ Labcorp, La Jolla, CA, United States; ^2^ PetDx, The Center for Novel Therapeutics, La Jolla, CA, United States

**Keywords:** cell-free (fetal) DNA, cfDNA (circulating cell free DNA), NIPT (non-invasive prenatal testing), 22q11.2 deletion syndrome, prenatal screening and diagnosis, 22q deletion syndrome

## Abstract

**Introduction:** Non-invasive prenatal screening (NIPS) *via* cell-free DNA (cfDNA) screens for fetal chromosome disorders using maternal plasma, including 22q11.2 deletion syndrome (22q11.2DS). While it is the commonest microdeletion syndrome and has potential implications for perinatal management, prenatal screening for 22q11.2DS carries some inherent technical, biological, and counseling challenges, including varying deletion sizes/locations, maternal 22q11.2 deletions, confirmatory test choice, and variable phenotype.

**Materials and methods:** This study addresses these considerations utilizing a retrospective cohort of 307 samples with screen-positive 22q11.2 NIPS results on a massively parallel sequencing (MPS) platform.

**Results:** Approximately half of the cases reported ultrasound findings at some point during pregnancy. In 63.2% of cases with diagnostic testing, observed positive predictive values were 90.7%–99.4%. cfDNA identified deletions ranging from <1 Mb to 3.55 Mb, with significant differences in confirmed fetal *versus* maternal deletion sizes; estimated cfDNA deletion size was highly concordant with microarray findings. Mosaicism ratio proved useful in predicting the origin of a deletion (fetal *versus* maternal). Prediction of deletion size, location, and origin may help guide confirmatory testing.

**Discussion:** The data shows that MPS-based NIPS can screen for 22q11.2DS with a high PPV, and that collaboration between the laboratory and clinicians allows consideration of additional metrics that may guide diagnostic testing and subsequent management.

## Introduction

Since the introduction of cell-free DNA (cfDNA) screening in 2011, significant progress has been made in the area of non-invasive prenatal screening. Current screening is moving beyond traditional cfDNA for the common aneuploidies, expanding in scope to genome-wide copy number variants (Lefkowitz et al., 2016; Van Opstal et al., 2018; van der Meij et al., 2019; Rafalko et al., 2021a; Soster et al., 2021; Wang et al., 2021) and single gene disorders (Zhang et al., 2019; Hoskovec et al., 2022; Mohan et al., 2022). Another area of expansion for cfDNA screening is the detection of select microdeletion syndromes. Of particular interest is screening for 22q11.2 deletion syndrome (22q11.2DS), a condition with a reported prevalence of one in 1,000 in unselected fetuses or one in 3,000–6,000 live births (McDonald-McGinn et al., 2015).

Also known as DiGeorge syndrome (OMIM #188400) or velocardiofacial syndrome (VCFS, OMIM #192430), 22q11.2DS is associated with a wide spectrum of anomalies, with cardiac defects, immune system dysfunction, oral clefting, hypocalcemia, developmental delays, and behavioral complications being among the most commonly reported features (McDonald-McGinn et al., 2015; Campbell et al., 2018). However, some of these features cannot be ascertained during the prenatal or even the neonatal period (e.g., developmental delays, immune dysfunction, behavioral complications, *etc.*) although cardiac anomalies, oral clefting, polyhydramnios, renal abnormalities, and skeletal abnormalities are among the findings that may be identified during routine ultrasonography (McDonald-McGinn et al., 1999). CfDNA screening for 22q11.2DS became clinically available in 2013 (Helgeson et al., 2015), yet most professional societies have yet to support routine screening for this condition during pregnancy due to a lack of clinical data and the perception of a reduced positive predictive value (PPV) associated with a positive screening result, ranging from 18% to greater than 97% (Helgeson et al., 2015; Gross et al., 2016; Martin et al., 2018; American College of Obstetricians and Gynecologists’ Committee on Practice Bulletins—Obstetrics et al., 2020; Bevilacqua et al., 2021; Soster et al., 2021; Dar et al., 2022; Dungan et al., 2022; Rose et al., 2022). Recently, the American College of Medical Genetics (ACMG) “suggests that screening for 22q11.2 deletion syndrome be offered to all patients” as a conditional recommendation based on moderate certainty of evidence (Dungan et al., 2022). Prenatal identification of 22q11.2DS allows parents to make informed reproductive choices and may have benefit for both perinatal management and maternal health, and may help to avoid a potential “diagnostic odyssey” for an affected child (McDonald-Mcginn et al., 2001; Bassett et al., 2011; Cheung et al., 2014; Fung et al., 2015; McDonald-McGinn et al., 2015; Van et al., 2016; McDonald-McGinn, 2018). Furthermore, 22q11.2DS often occurs *de novo*, is not associated with maternal age, and affected fetuses may or may not present with ultrasound findings; these features suggest that many cases of 22q11.2DS may not otherwise be identified in the prenatal or neonatal period *via* ultrasound or routine assessment of the patient’s family or obstetric history (McDonald-McGinn et al., 2015).

Screening for 22q11.2DS by cfDNA is accompanied by inherent biological and technical challenges. A variety of deletion sizes have been reported with 22q11.2DS owing to the low copy number repeats (LCRs), labeled A through D, in the area (McDonald-McGinn et al., 2015; Campbell et al., 2018). Approximately 85% of patients have a ∼2.54 Mb sized deletion, which had been frequently described as a “3 Mb” deletion, from A-D LCRs (McDonald-McGinn et al., 2015). The remaining ∼15% have smaller atypical or nested deletions (for example, involving the A-B LCRs or C-D LCRs) which may be associated with a milder phenotype and reduced penetrance (McDonald-McGinn et al., 2015). This region is particularly susceptible to meiotic error due to the significant homology between these LCRs; non-allelic homologous recombination leads to both the “3 Mb” deletion as well as the smaller, nested deletions (McDonald-McGinn et al., 2015). Given the size of 22q11.2 deletions, even the typical “3 Mb” deletion will be below the resolution of detection by routine prenatal chromosome analysis; diagnostic confirmation of 22q11.2 deletions typically requires microarray and/or fluorescent *in situ* hybridization (FISH). Additionally, common FISH probes for detection of 22q11.2 deletions used by diagnostic laboratories may include N25, *TUPLE1/HIRA* and *TBX1* (McDonald-McGinn et al., 2015; McDonald-McGinn, 2018). These probes generally hybridize between the A-B LCRs, a region included in the typical “3 Mb” deletion (McDonald-McGinn et al., 2015; McDonald-McGinn, 2018). However, use of these FISH probes for the detection of atypical nested deletions may have limited clinical utility as the deletion may be proximal or distal to the FISH probes used.

Approximately 10% of affected individuals inherit the deletion from an affected parent, although this percentage is significantly higher (60%) for nested/smaller atypical deletions (McDonald-McGinn et al., 1999; Bassett et al., 2011; McDonald-McGinn et al., 2015). CfDNA screening utilizes maternal plasma containing both fetal (placental) and maternal cfDNA, therefore, presence of a maternal CNV in a region of interest precludes fetal assessment of that particular region. Even though fetal assessment using cfDNA may not be possible when a maternal CNV is suspected, detection and reporting of a suspected maternal 22q11.2 deletion suggests a 50% risk for an affected fetus. These factors underline both the benefits and limitations of this screening test, and support the role of diagnostic confirmation as the next most reasonable step. Any patient with a screen positive result should be referred for genetic counseling, with a discussion on the option of diagnostic testing.

The current study addresses these clinical and laboratory considerations following a screen-positive cfDNA result by analyzing a cohort of 307 samples where a 22q11.2 deletion (either fetal or suspected maternal) was detected *via* cfDNA screening at a single commercial laboratory, including an analysis of observed PPVs in a retrospective cohort of patients that pursued diagnostic testing. This study also examines how massively parallel sequencing (MPS)-based cfDNA screening is capable of predicting maternal *versus* fetal events involving the 22q11.2 region, the implications of the presence of ultrasound findings and/or maternal phenotype, the predicted sizes and locations of 22q11.2 deletions on cfDNA as compared to predicted size/location on diagnostic testing in the cohort, and considerations for follow-up diagnostic testing following a screen positive result.

## Materials and methods

Maternal blood samples were submitted from November 2013 through August 2021 for cell-free DNA screening with analysis of 22q11.2 deletion syndrome using either “traditional” cfDNA screening with microdeletion analysis (MaterniT^®^ 21 PLUS with Enhanced Sequencing Series) or genome-wide cfDNA screening (MaterniT^®^ GENOME) as selected by the ordering provider. MaterniT^®^ 21 PLUS screens for trisomies of chromosomes 21, 18, and 13 with the option of expanded content (sex chromosome aneuploidies, and the Enhanced Sequencing Series which includes trisomies 16 and 22 as well as microdeletions associated with 1p36 deletion, Wolf–Hirschhorn, Cri-du-chat, Langer–Giedion, Jacobsen, Prader–Willi, Angelman, and DiGeorge syndromes). MaterniT^®^ GENOME screens for aneuploidy of any chromosome, CNVs ≥7 Mb in size as well as the select microdeletions <7 Mb in size mentioned above. During the time frame, nearly 850,000 samples were screened for 22q11.2 deletion syndrome across both assays. Blood samples were subjected to DNA extraction, library preparation, and genome-wide MPS as previously described (Jensen et al., 2013) with detection of subchromosomal CNVs as described by Zhao et al. (2015). For cases submitted for genome-wide analysis, sequencing data were analyzed using a proprietary algorithm to detect aneuploidies and other subchromosomal events as described by Lefkowitz et al. (2016). Pretest and posttest counseling and informed consent were the responsibility of the clinicians ordering the testing.

All clinical cfDNA specimens which detected suspected fetal and suspected maternal 22q11.2 deletions were compiled, along with all available diagnostic outcomes. Diagnostic outcomes were obtained from two sources. First, outcome information was collected, when available, from the ordering provider. Second, positive cfDNA samples were cross-referenced with diagnostic results (FISH, microarray, and karyotype) submitted to Labcorp from chorionic villus, amniocentesis, neonatal and/or maternal peripheral blood, and products of conception specimens during a corresponding timeframe. The process of consolidation and comparison of data across the datasets (cfDNA results and microarray results) was approved by Aspire IRB under clinical protocol SCMM-RND-402. For a cfDNA sample to be considered a match to a microarray specimen, the diagnostic and screening results were required to have identical patient identifiers (name and date of birth), and the collection date for the diagnostic test had to be within 90 days of the patient’s cfDNA screening date. Cases outside the 90-day window were adjudicated to ensure the screening and diagnostic results were from the same pregnancy. However, matched diagnostic testing on a maternal blood specimen could have occurred at any time and was not limited to the 90-day window. Because postnatal testing on the neonate is typically under a different set of identifiers (typically the name and birth date of the neonate), matching of those samples back to the cfDNA specimen was not possible in most cases. Obstetric pregnancy outcome and postnatal follow-up were rarely available and thus are not analyzed in the study. A cfDNA result was classified as a “true positive” for 22q11.2 deletion syndrome when the deletion was confirmed *via* FISH testing and/or microarray analysis. A “false positive” classification was assigned when the abnormal screening result was not confirmed by diagnostic testing. Cases were considered to have “incomplete” diagnostic testing when only FISH or karyotype were normal, with no microarray ordered, or when a maternal deletion was suspected based on the cfDNA sequencing data and only fetal testing was performed, or *vice versa* (fetal deletion was suspected based on the cfDNA sequencing data and only maternal testing was performed). Such cases were not treated as false positives, as the completed testing was inadequate to rule out the presence of a 22q11.2 microdeletion. However, PPV calculations were performed in multiple ways, including a version where these were treated as hypothetical false positives for the most conservative estimation of PPV.

Beginning in September of 2015, secondary to bioinformatics and assay enhancements, additional data for screen-positive 22q11.2 deletion specimens included the estimated deletion size, estimated breakpoints, and the event specific fraction on cfDNA allowing for the calculation of mosaicism ratio. Mosaicism ratio (MR) is a laboratory metric derived by dividing the fraction of cfDNA associated with the abnormal event by the overall fetal fraction of the specimen, as described by Rafalko et al. (2021b). Cases with a disproportionally high MR (in consideration with other sequencing metrics, such as a considerably elevated z-score), were assigned as “likely maternal” for reporting by the laboratory director. A proprietary algorithm is used to flag events that are “likely maternal,” but the laboratory director has the final discretion to determine how to report the event in context of the rest of the sequencing data. In these circumstances, patient results were reported as screen positive for 22q11.2 DS with an additional “likely maternal” comment and also noted assessment of fetal status for the 22q11.2 region was precluded. Cases without this additional comment on the patient report were designated “suspected fetal” for the purpose of the current study.

Maternal demographics and indications for testing were recorded as provided by the ordering clinician at the time of testing on the test requisition form (TRF). TRFs were reviewed for additional details about ultrasound findings and/or family history, where indicated; the laboratory’s clinical database used for documenting client discussions and tracking outcomes was also reviewed for additional notes from the provider about ultrasound findings, family history, pregnancy outcomes, and any other relevant case details. These demographics and details about test indications were compiled and analyzed.

Study data was statistically described using counts, rates, and measures of central tendency. Positive predictive value with 95% confidence interval was calculated using the VassarStats Website for Statistical Computation. Deletion sizes and mosaicism ratios were compared using Wilcoxon rank-sum tests. A Fisher’s exact test was used to test for an association between ultrasound findings and fetal diagnostic testing. Concordance between cfDNA-predicted deletion size and microarray-confirmed deletion size was plotted and fitted with a linear regression line. For all calculations, *p*-values less than 0.05 were considered statistically significant. Statistical analyses and generation of plots and figures were performed using R version 4.0.5 and the dplyr, ggplot2, lubridate, stringr, ggalt, scales, and ggpubr packages.

Collection of outcomes was approved by AspireIRB under clinical protocol SCMM-RND-402 and all clinical data was de-identified. Informed consent was not required as AspireIRB declared that this research meets the requirements for a waiver of consent under 45 CFR 46 116(f)[2018 Requirements].

## Results

### Study cohort

There were 307 cases with 22q11.2 deletions reported either as suspected maternal (precluding fetal assessment) (57.7%, n = 177) or suspected fetal (42.3%, n = 130) from cfDNA screening through August 2021. Most cases were identified *via* the standard cfDNA assay with select microdeletions opted-in (64.2%, n = 197), while the remainder were identified *via* the genome-wide assay (35.8%, n = 110). Nearly all cases were singleton pregnancies (99.3%, n = 305), with 2 cases of twin pregnancies (0.7%). The median maternal age was 29.0 years (range 15–45 years) with a median gestational age of 19 weeks (range 9–36 weeks). The median fetal fraction was 10.48% (range 2.96%–41.18).

**FIGURE 1:**
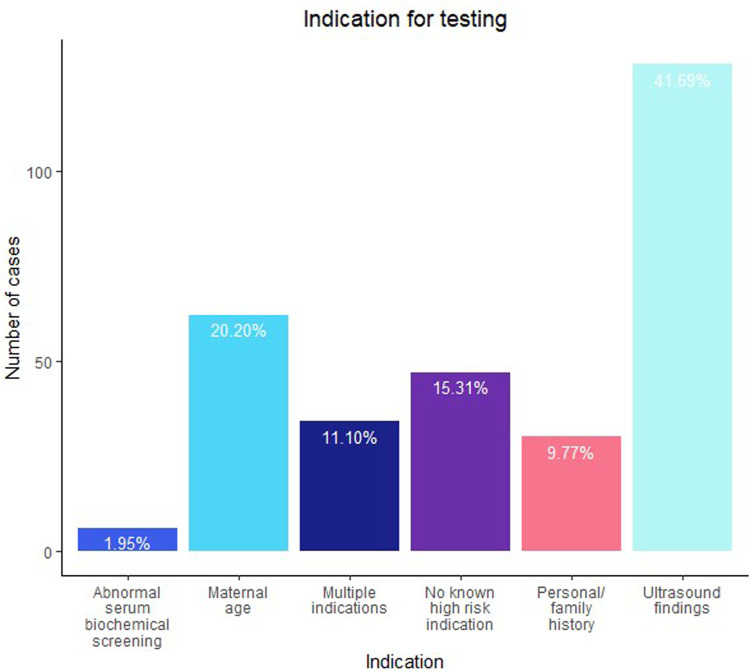
Indication for testing, as provided on the test requisition form.

Figure 1 shows the indications for testing. The most common indication for testing was ultrasound findings (41.7%, n = 128), followed by maternal age (20.2%, n = 62) and no known high-risk indication (15.3%, n = 47). Of note, 34 cases were referred with multiple indications for testing; 23 of those included ultrasound findings as one of the reasons. When outcomes were collected, an additional 6 cases were noted to later have ultrasound findings in the outcome relayed by the provider or noted on the TRF submitted with a diagnostic specimen. Ultimately, 157 cases (51.1%) had an ultrasound finding reported either at the time of testing or at some point during the pregnancy. The details of the ultrasound findings, where available, are further discussed later. For 36/177 of the suspected maternal cases, notes or comments about maternal phenotype (or lack thereof) potentially associated with 22q11.2DS were available; a maternal phenotype was noted in 24 cases and absence of any phenotype was noted in 12 cases.

### cfDNA results

**FIGURE 2:**
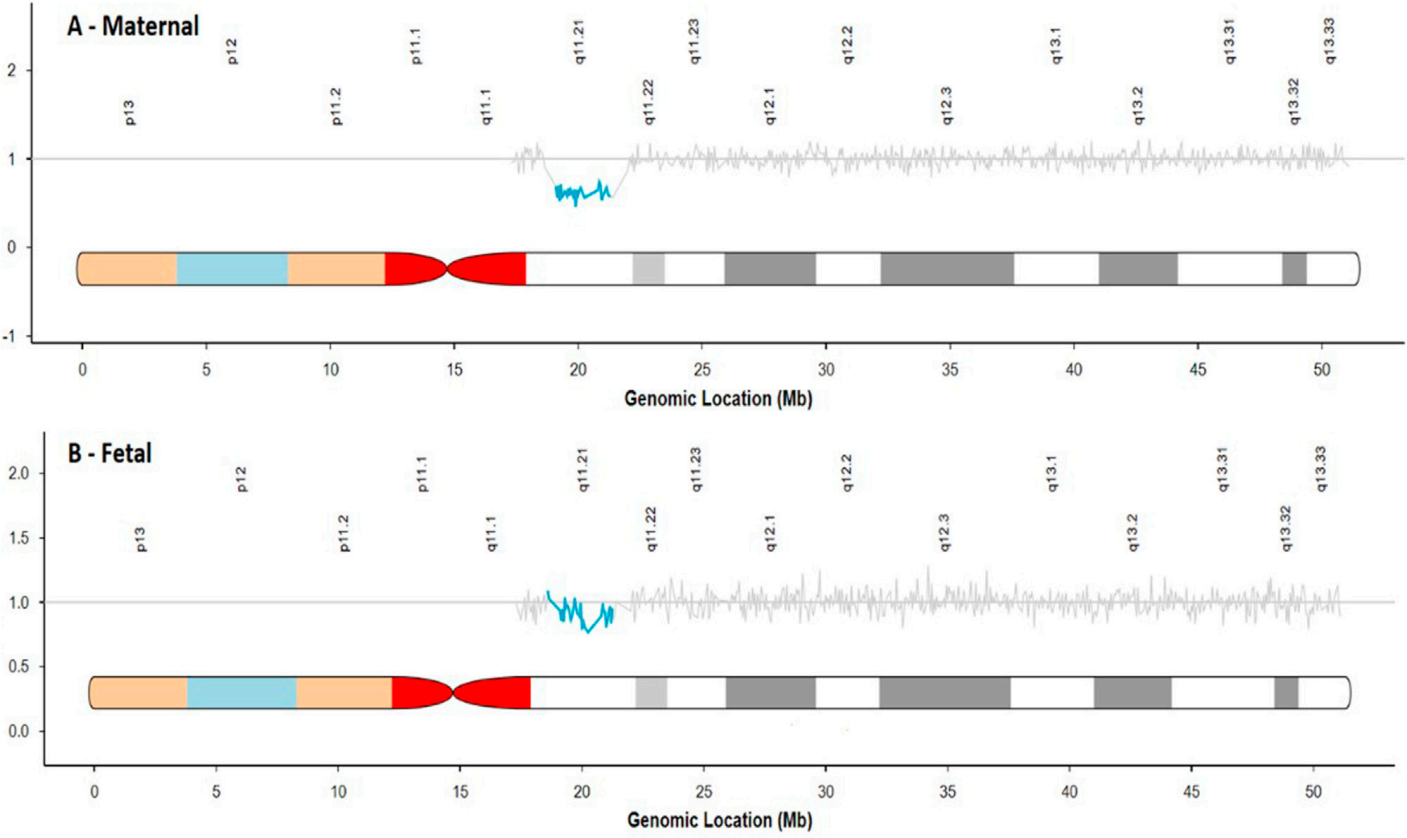
Comparison of sequencing traces for a suspected maternal deletion **(A)** and suspected fetal deletion **(B)**.

Of the 307 suspected deletions, 177 were suspected to be maternal in origin, while the remaining 130 appeared to be fetal in origin based on the cfDNA sequencing data. Figure 2 shows an example of the sequencing data for both a suspected maternal deletion and fetal deletion in the region of 22q11.2.

**FIGURE 3:**
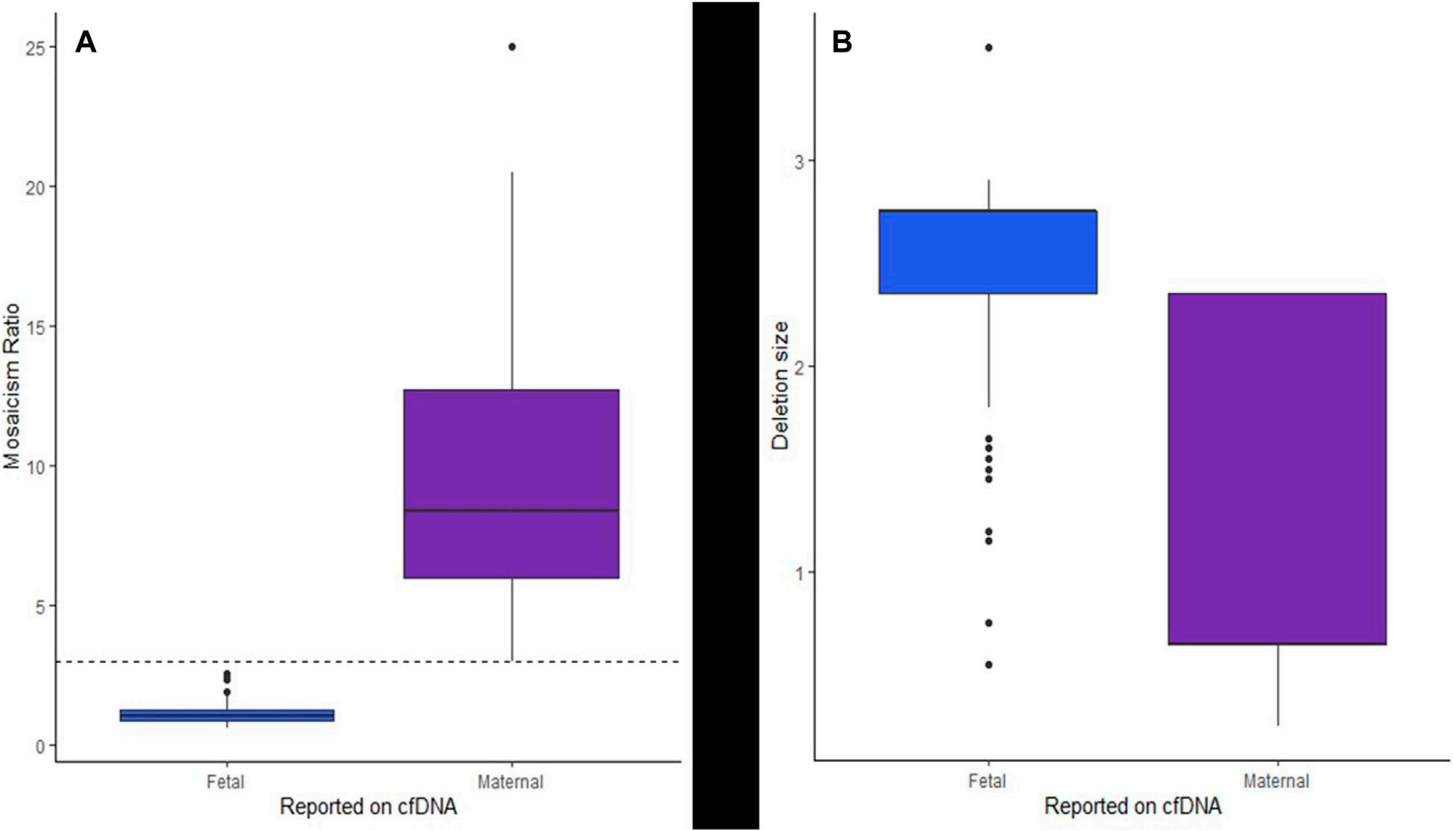
Box and whisker plots showing **(A)** the separation of the MR of suspected fetal and suspected maternal cases from cfDNA and **(B)** the estimated deletion sizes for suspected fetal events as compared to suspected maternal events from cfDNA.

A mosaicism ratio (comparing the fraction of cfDNA harboring the 22q11.2 deletion to the overall fetal fraction) was available for 241 cases (early assay versions did not include this metric). Figure 3A shows the near-complete separation of MRs between the suspected fetal and suspected maternal cases, with a statistically significant difference in the MR for the suspected fetal *versus* suspected maternal cases (*p* < 2.2e-16). MR in maternal cases was nearly always above three and MR in fetal cases was nearly always less than 3, with median MR in suspected fetal cases of 1.023 and median MR in suspected maternal cases of 8.354.

The cfDNA sequencing data provided an estimated deletion size and predicted breakpoints for 237 cases. (early version of the assay did not predict deletion sizes and breakpoints). Table 1 shows the details of the predicted deletion sizes from cfDNA overall, as well as for suspected maternal deletions and suspected fetal deletions. Supplementary Figure S1 shows a plot of the start and end breakpoints predicted from cfDNA, with groupings of frequent deletions of similar sizes, the common, larger deletions toward the bottom of the figure and the smaller nested/atypical deletions toward the top. The star on the *x*-axis indicates the approximate location of most commercially available FISH probes (N25, *TUPLE1/HIRA, TBX1*). There was a statistically significant difference (*p* < 2.2e-16) in the predicted sizes between the suspected maternal deletions and suspected fetal deletions. Fetal deletions tended to be larger, while maternal deletions tended to be smaller. Figure 3B shows a plot of the estimated deletion sizes by suspected maternal *versus* fetal origin from cfDNA.

**TABLE 1:** Estimated 22q11.2 deletion sizes from cfDNA.

	Median (Mb)	interquartile range (Mb)	Range (Mb)
All deletions (n = 237)	2.35	0.65–2.35	0.25–3.55
Suspected fetal (n = 99)	2.75	2.35–2.75	0.55–3.55
Suspected maternal (n = 138)	0.65	0.65–2.35	0.25–2.35

Mb, megabases.

Deletion sizes from cfDNA were then evaluated in the context of ultrasound findings as shown in Supplementary Figure S2. When suspected maternal cases were removed (which as noted, tend to be smaller), and only suspected fetal cases were analyzed (Supplementary Figure S2B, there was no significant different in the size of deletions with and without ultrasound findings (*p* = 0.6687). Analysis of only confirmed fetal cases (Supplementary Figure S2C) was also not significant (*p* = 0.2588). Initial analysis of the overall cohort (Supplementary Figure S2A), before removal of the suspected maternal cases, showed the appearance of a significant difference in deletion size (*p* = 6.533e-10), which was clearly being skewed by the maternal cases.

Based on the overall cohort there appeared to be a significant difference in the size of the deletions in cases with and without ultrasound findings (*p* = 6.533e-10); however, when the suspected maternal cases (which as noted above, tend to be smaller) were removed, and only suspected fetal cases were analyzed, there was no significant difference (*p* = 0.6687).

### Diagnostic testing and positive predictive value

There were 194 cases with a diagnostic test result available (63.2%). Of the 194 cases with diagnostic testing results available, 83 had only fetal testing, 76 had only maternal testing, and 35 had testing for both. Cases with fetal ultrasound findings were significantly more likely (3.45x) to have a fetal diagnostic test than cases without ultrasound (*p* = 3.889e-07, OR = 3.45, 95% CI: 2.07–5.83). There were 17 cases that were considered to have incomplete diagnostic testing, such as only karyotype; the details of these 17 cases are described in Supplementary Table S1 This leaves 177 cases with complete diagnostic testing for calculation of positive predictive values. If all 177 cases were considered for a PPV calculation (both suspected maternal (n = 102) and fetal (n = 75), with confirmation on either fetal testing or maternal testing), the PPV is 99.4% (95% CI: 96.4%–99.9%). If only fetal cases are considered (n = 75; suspected fetal cases with fetal testing available) the PPV of the assay is 98.7% (95% CI: 91.8%–99.9%). For the most conservative estimate of PPV in the cases with diagnostic testing, if the 17 “incomplete” cases were all considered “false positives,” the PPV is 90.7% (95% CI: 85.5%–94.2%). Supplementary Table S2 summarizes these PPV calculations. Supplementary Figure S3 shows a flow chart that summarizes the diagnostic testing for the 194 cases.

If the cases without diagnostic testing were factored into the PPV calculations, an upper and lower bound to PPV can be calculated as included in Supplementary Table S2. If all cases without complete diagnostic testing were included and treated as true positives, the upper bound to the PPV would be 99.7%. If all cases without complete diagnostic testing were treated as false positives, the lower bound to the PPV would be 57.3%.

There was one false positive case in a patient screened at 12 weeks of gestation with the indication of advanced maternal age in a singleton pregnancy. The data suggested a fetal deletion, with an overall fetal fraction of 4.43%, an MR of 1.496, and an estimated deletion size of 2.75 Mb on cfDNA. FISH and microarray *via* amniocentesis were reportedly normal; no maternal testing was completed.

Supplementary Table S3 summarizes the diagnostic testing available for this cohort. As seen in the supplement, the most common assay/specimen combinations were maternal blood microarray (n = 58), amniotic fluid microarray (n = 35), and postnatal/neonatal microarray (n = 31). Of the cases with fetal/neonatal testing (n = 118), 45.8% (n = 54) deferred testing to the postnatal period or on a POC specimen following a loss.

**FIGURE 4:**
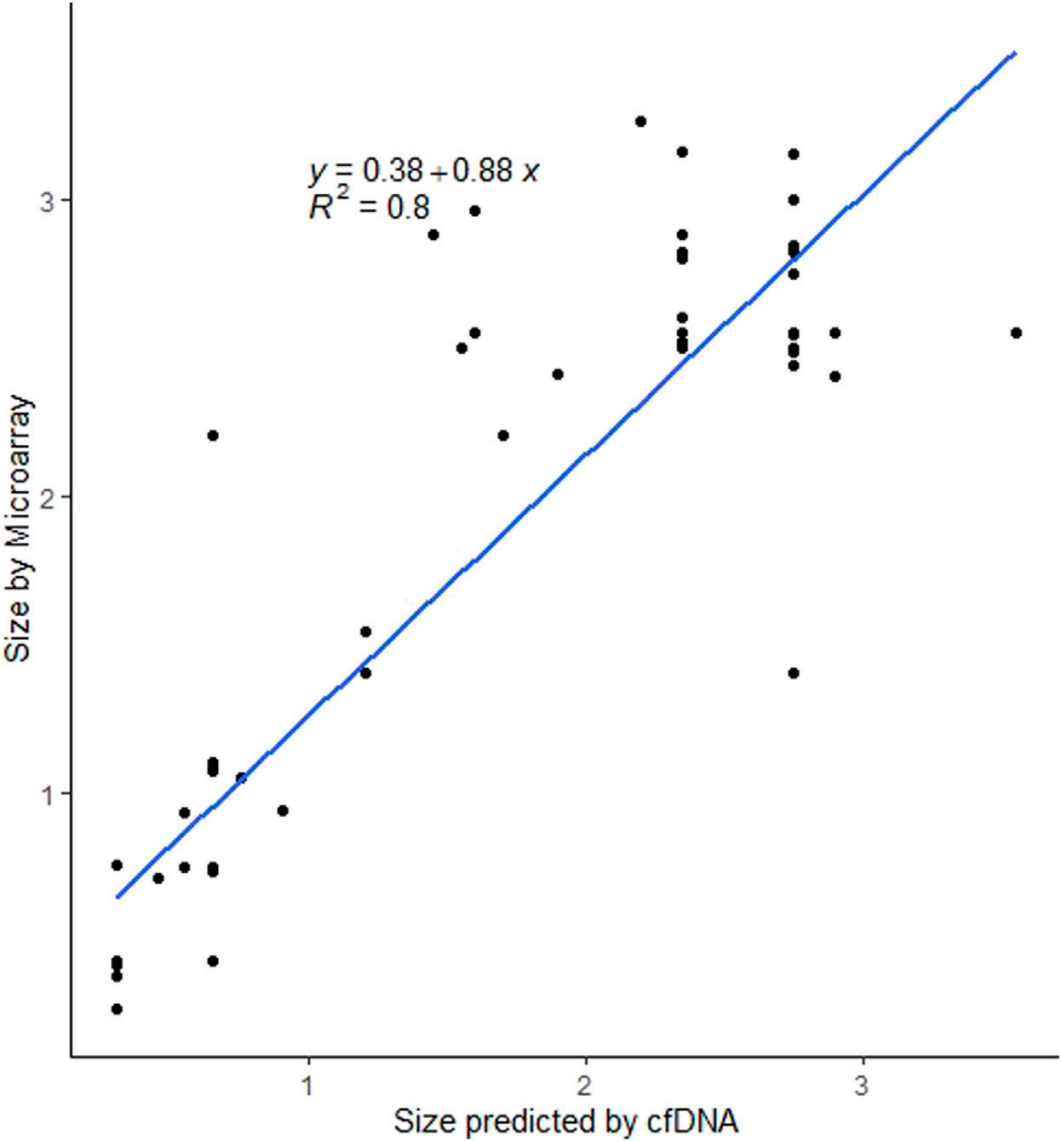
Scatterplot showing the relationship between estimated deletion size on cfDNA and confirmed deletion size on microarray.

For the 89 cases with deletion sizes available from microarray confirmation, the median deletion size was 2.48 Mb (range 0.268–3.26 Mb). Predicted fetal confirmed deletions (n = 37) had a median size of 2.55 Mb (range 0.93–3.26 Mb) and predicted maternal confirmed deletions (n = 52) had a median size of 0.75 Mb (range 0.268–3.160 Mb). Similar to the predicted sizes by cfDNA, there is a significant difference (*p* = 2.728e-06) in the size of deletions on array for those that were predicted to be maternal events *versus* fetal events from cfDNA. For cases with a predicted size available from cfDNA and confirmed size available from microarray, Figure 4 shows the relationship between the estimated size and the actual size. Using linear regression, sizes were highly concordant between array and cfDNA with an r-square of 0.8.

When a deletion of fetal origin was suspected (n = 130), complete fetal testing was available for 75 cases. Of those, 74 had a fetal deletion confirmed; the other was a false positive with no maternal testing. Four of the suspected fetal cases with diagnostic confirmation also had maternal results available, all of which were normal. One of these “fetal” cases was noted to be paternally inherited based on a known deletion in the family.

When a maternal deletion was suspected (n = 177), testing was available for 105 cases, however, complete maternal testing was available for 97 cases (an additional eight had “incomplete” maternal testing). Of those 97, a maternal deletion was confirmed in all cases. Of note, of the 97 cases with a confirmed maternal deletion, 35 had knowledge of the maternal deletion prior to testing, while 63.9% presumably did not based on the outcome collection notes. Of these 35 cases, two reported a maternal phenotype. The remaining 33 did not report a maternal phenotype to the laboratory, although this does not preclude the presence of findings that were simply not reported to the lab. The remaining 22 cases with a maternal phenotype reported were presumably not known to have a 22q11.2DS prior to testing.

Additionally, there were 43 suspected maternal cases that had fetal testing available (31/35 from the “testing in both” group and 12/83 from the “fetal testing only” group). Of those 43 cases, a fetal deletion was found in 58.1% cases (n = 25, 5/12 from the “fetal testing only” group and 20/31 from the “testing in both” group), confirming fetal inheritance of the suspected maternal deletion. For the 113 cases without diagnostic testing, Supplementary Table S6 summarizes the indications for testing and provides details about ultrasound findings, where available, as well as any notes about pregnancy outcome. Similar to the overall cohort, approximately half of cases without diagnostic testing had ultrasound findings noted.

### Ultrasound findings

As noted above, 157 cases (51.14%) were noted to have ultrasound findings either at the time of testing or at some point during the pregnancy. Although the degree of detail regarding the specific ultrasound findings varied (for example, 19 cases had no further details about the ultrasound findings), most cases contained some additional information about the findings. Supplementary Table S4 contains the summary details of the ultrasound findings across these 157 cases, as available. Supplementary Table S7 lists the 75 suspected fetal cases with diagnostic testing (74 true positives and one false positive) with the ultrasound finding information amended.

Cardiac findings were by far the most frequent anomaly reported on ultrasound (95/157, 60.5%), with 71 isolated cardiac anomalies, and 24 cases of cardiac anomalies accompanied by extracardiac anomalies. The most commonly reported cardiac anomalies were Tetralogy of Fallot (TOF), ventricular septal defect (VSD), and truncus arteriosus. Complex cardiac anomalies were reported in isolation in 15 cases and with other extracardiac anomalies in 2 cases. Unspecified cardiac anomalies were reported in isolation in 20 cases and with other extracardiac anomalies in 10 cases. A handful of other specific cardiac anomalies were reported, as detailed in Supplementary Table S5. There were 13 cases of reported renal findings on ultrasound; six of those were seen in conjunction with cardiac anomalies. Abnormalities of amniotic fluid were reported in a group of cases; polyhydramnios was seen as the only finding in 4 cases and in conjunction with other findings in 8 cases; oligo/anhydramnios was reported in 2 cases. Fetal growth concerns, either growth restriction (n = 13) or shortened long bones (n = 1), were reported frequently, as well. Oral clefts were only seen in two cases.

## Discussion

The current study reflects on a number of considerations for prenatal screening for 22q11.2DS by cfDNA *via* massively parallel sequencing, as observed through the lens of the clinical experience of a single laboratory. These considerations are important for patient counseling prior to 22q11.2DS screening, but are especially critical for patients who receive a screen positive result.

In this cohort, the PPVs of approximately 91%–99% were consistent with those reported for the MPS methodology (Helgeson et al., 2015; Liang et al., 2019; Soster et al., 2021) and higher than those reported by SNP-based cfDNA methodologies of approximately 16% under the original protocol to 53% in a more recent study (Martin et al., 2018; Dar et al., 2022); although Dar et al. had diagnostic testing data on a higher percentage of screen positive cases, this study includes a larger number of screen positive cases with diagnostic testing. Another study using a targeted methodology reported no false positive cases for 22q11.2DS (Bevilacqua et al., 2021). The higher PPVs seen in the current study may, in part, be influenced by the presence of ultrasound findings in roughly half of all screen positive cases, and the clear association between the presence of ultrasound findings and the likelihood of fetal diagnostic testing demonstrated in this cohort. Patients with ultrasound findings at any point in the pregnancy were significantly more likely to have fetal diagnostic testing results available. For patients with structural anomalies on ultrasound, professional societies recommend diagnostic testing with microarray (Practice Bulletin, 2016; American College of Obstetricians and Gynecologists’ Committee on Practice Bulletins—Obstetrics et al., 2020), but some patients may be reticent and opt to pursue screening. Screening is not a substitute for diagnostic testing. Nearly half of patients in this cohort who pursued fetal/neonatal diagnostic testing deferred this testing until after the baby was delivered or until after a pregnancy loss.

While PPV is one important metric, there are a number of other important factors that should be considered after a screen positive result. Laboratory algorithms, such as MR, may be useful in predicting whether the deletion observed in the cfDNA sequencing data is more likely to be of maternal or fetal origin. Indeed, in this study, the proprietary algorithm (informed by the strength of the sequencing signal of the event as well as other metrics such as MR, Z-score, and estimated fetal fraction) was helpful in predicting whether the event was maternal *versus* fetal in origin. Assignment of maternal *versus* fetal origin on cfDNA was accurate in all true positive cases. Using MR alone with a cutoff of three would correctly classify the majority of cases as maternal or fetal, although one confirmed maternal case had an MR of 2.969. This pregnancy was tested at 34 weeks gestation and had a fetal fraction of 27.83%, which could have influenced the “affected” fraction and diminished the effect of the maternal contribution. Prediction of a maternal event could help guide testing for the pregnant patient, if desired, and if confirmed, would convey a 50% risk for fetal inheritance. Of the maternal deletions with fetal testing, approximately 50% of cases theoretically should confirm a fetal deletion; in this study, we observed that ∼58% of fetuses inherited the maternal deletion. This is likely enriched by cases with ultrasound findings, which were more likely to pursue fetal testing.

For some patients, incidental discovery of a previously unknown 22q11.2 deletion may be helpful in explaining symptoms or a previously unrecognized phenotype, while for others, this information may be unwelcome or anxiety producing. As seen in this cohort, of the 24 cases with a reported maternal phenotype, 22 were not known/reported to the lab to have a known 22q11.2 deletion. Conversely, for the 35 confirmed maternal cases known prior to cfDNA screening, a phenotype was only reported to the lab for two cases. Given the retrospective nature of this study, we cannot assume an absence of maternal phenotype in cases where one was not reported. There were only 12 suspected maternal cases were lack of a maternal phenotype was confirmed by the provider. Pretest counseling for cfDNA, especially when 22q11.2DS is included, should include a discussion of the risks, benefits and limitations of testing, including the risk for false positives and false negatives, and the possibility of uncovering unexpected maternal findings. In this study, limited information about maternal phenotype was available; results around maternal phenotype in this study should be interpreted with caution and additional information about maternal phenotypes would be useful in further exploring the impact of these findings. This affords an area for future study. It should be noted that approximately one-third of confirmed maternal cases in the current study were already known to the patient prior to undergoing cfDNA screening. Though the maternal 22q11.2 event precluded assessment of the fetus for that specific region, these patients were still able to receive unhindered fetal screening for the remainder of the conditions assessed by the assay.

cfDNA screening using MPS can identify a range of deletion sizes for 22q11.2DS and is able to estimate the size and predicted breakpoints of the deletion, as demonstrated in this study. In fact, the data from the cohort with cfDNA-predicted deletion size and microarray-confirmed deletion size showed that cfDNA estimates were highly concordant with the diagnostic confirmation. Though the size and breakpoints of 22q11.2 deletions predicted by MPS-based cfDNA closely align with diagnostic results, these data points should be considered estimates, as MPS groups cfDNA into 50 kb “bins”; this grouping of cfDNA by genomic regions means that a margin of error will exist for all size and breakpoint predictions (Bassett et al., 2011; Lefkowitz et al., 2016). Diagnostic testing with microarray analysis is essential for confirmation of deletion size and for determining the gene content in the deleted region. In general, maternal deletions were significantly smaller than fetal deletions, both in predicted and actual size. However, for both maternal and fetal events, the smallest confirmed deletion was less than 1 Mb. Given the genotype-phenotype associations, this could be relevant for patient counseling (McDonald-McGinn et al., 2015).

cfDNA size/breakpoint estimation also has practical implications for choosing an appropriate follow-up test; atypical or nested deletions may not be confirmed by FISH testing and 22q11.2DS deletions are typically below the resolution of a karyotype. In this cohort, there were at least 5 cases in which FISH testing was reportedly negative but a deletion was found on microarray. Most commercial FISH probes (N25, *TUPLE1/HIRA*, and *TBX1*) hybridize between low copy number repeat sequences A and B, so deletions that do not span this region will not be detected using these typical FISH probes (McDonald-McGinn et al., 1999; McDonald-McGinn et al., 2015; McDonald-McGinn, 2018). As seen in Supplementary Figure S1, while most cases were predicted to span the region that would be captured by these FISH probes, many of the smaller nested/atypical deletions were not predicted to overlap the hybridization region for these probes. Use of microarray for diagnostic confirmation of cfDNA results is recommended to avoid false negative results that may occur from FISH testing.

Additionally, microarray may also uncover findings beyond the 22q11.2 deletion suggested by cfDNA. It is worth noting that 16 of the cases with microarray in this cohort reported an additional finding beyond the suspected 22q11.2 deletions. Most of these findings were on maternal arrays; details of the additional array findings are provided in Supplementary Table S5. These additional findings, which would have been missed by FISH testing alone, may have clinical implications for the patient and/or fetus. There were also five cases with a negative FISH but a positive array, including one case with a deletion found on maternal array for which postnatal FISH was “incorrectly” ordered for the baby (the maternal deletion would not be detected by the FISH probes utilized). To the provider’s knowledge, this patient did not return for follow-up postnatal microarray for the child.

Lastly, cardiac anomalies were especially prevalent in this cohort, as expected. Oral clefting was only seen in two cases, but may be limited by the ability of ultrasound to detect palatal anomalies especially those isolated to the soft palate. One study estimates the sensitivity for prenatal diagnosis of cleft lip/palate to be 88% *via* evaluation of the upper lip and notes that the overall sensitivity of ultrasound to diagnosis oral clefting is depressed by lower detection rates of isolated cleft palate (Maarse et al., 2011).

### Limitations

This was a retrospective study of data available to the laboratory and thus represents an incomplete picture of the outcomes for these cases. Ultrasound finding details may be underreported or incomplete, as these details were primarily derived from the test requisition form. Diagnostic testing results were not available for nearly 40% of screen-positive cases and availability of fetal diagnostic testing results showed an association with the presence of ultrasound findings, which may bias the data presented in this study, particularly the PPV. Stratification of the PPV calculation into subgroups, such as comparing PPV in patients with and without ultrasound findings, would not provide additional value in this study, as there was only one false positive reported in the cohort. Further studies could explore the PPV in a general obstetric population using a similar platform. Furthermore, even for cases with diagnostic testing results available, there may be additional testing performed that was not communicated or available for review. Limited information about obstetric and maternal outcomes was available and thus was not included in the study.

Identification and reporting of maternal and fetal events was also influenced by the technical limitations of the assay; in general, the assay is expected to detect maternal deletions more readily than fetal deletions since the majority of the cfDNA in a sample is maternally-derived. Very small fetal deletions, especially at lower fetal fractions, may be below the limit of detection for the assay. As such, the data comparing maternal and fetal deletion sizes may be skewed by the fact that smaller maternal deletions will be more readily detected than smaller fetal deletions.

Although many cases in this study had maternal testing reported, very few had details on paternal follow-up testing. There was one case which was known to be paternally inherited. Given the limited follow-up information for parental testing, it is not possible to estimate how many fetal cases were *de novo versus* inherited.

This study did not explore sensitivity, specificity, and NPV, as limited data is available on the screen negative cases, precluding reliable performance calculations. Robust collection of outcomes in screen negative cases was limited not only by the sheer number of screen negative cases, but also because many patients with a screen negative result may not pursue additional testing during the pregnancy, especially in the absence of ultrasound findings.

## Conclusion

When cfDNA screening is positive for 22q11.2 deletion syndrome, clinicians and patients are faced with several decisions about follow-up testing. Should testing involve just the fetus, or should the mother be evaluated, as well? What type of analysis is most appropriate on the diagnostic specimen? Will FISH be able to detect the predicted abnormality, or is microarray necessary? Data from massively parallel sequencing may help to answer these questions and guide appropriate follow up testing for screen positive patients.

Ultimately, this study shows that MPS-based cfDNA screening for 22q11.2DS can be an effective screening tool, may distinguish maternal from fetal events, and can be used to estimate the size and predicted location of the deletion. Positive screening results should be confirmed by diagnostic testing with microarray analysis, and no irreversible management decisions should be made on the basis of screening results alone, consistent with professional society recommendations (American College of Obstetricians and Gynecologists’ Committee on Practice Bulletins—Obstetrics et al., 2020; Dungan et al., 2022). Furthermore, counseling following a screen-positive result should include a discussion of the variability in clinical features, although there is some genotype-phenotype correlation noted (McDonald-McGinn et al., 2015). Indeed, even within this cohort, affected pregnant patients were discovered following prenatal screening who had not previously been identified. Collaboration and communication between the cfDNA screening laboratory and the healthcare provider, especially in the event of a screen positive result, can optimize follow-up care. Genetic counselors working within the laboratory can review the metrics reviewed in this study, such as predicted size and breakpoints, whether the sequencing data suggests a maternal or fetal event, and retrospective PPVs based on the laboratory’s experience and considering the patient’s phenotype or the presence of ultrasound findings. Given that 22q11.2DS is a relatively common chromosome condition and may have implications for the pregnant person and the fetus, the potential benefits to screening may outweigh the challenges in the setting of education and informed consent (McDonald-Mcginn et al., 2001; Bassett et al., 2011; Cheung et al., 2014; Fung et al., 2015; McDonald-McGinn et al., 2015; Van et al., 2016). As eloquently summarized by McDonald-McGinn et al., “Prenatal detection of 22q11.2DS [enables] future parents to make informed choices, prepare for obstetrical and neonatal management, and provide the opportunity to improve survival and outcome” (McDonald-McGinn et al., 2015).

## Data Availability

The datasets for this article are not publicly available due to concerns regarding participant/patient anonymity. Requests to access the datasets should be directed to the corresponding author.
