# Supplementary material for: Positive cfDNA screening results for 22q11.2 deletion syndrome—Clinical and laboratory considerations
Source: Front Genet. 2023 Mar 10;14:1146669. doi: 10.3389/fgene.2023.1146669 (PMC10036386; doi:10.3389/fgene.2023.1146669)
Supplement: Supplementary file 1 [file DataSheet1.pdf]

Supplementary Material

Positive cfDNA screening results for 22q11.2 deletion syndrome – clinical and laboratory considerations

Erica Soster[1*]†, Brittany Dyr[1]†, Jill Rafalko[1,2], Eyad Almasri[1], Philip Cacheris[1]

*** Correspondence:** Erica Soster: sostere@labcorp.com

1 Supplementary Data

Supplemental Figure 1 – Estimated breakpoints from cfDNA sequencing data

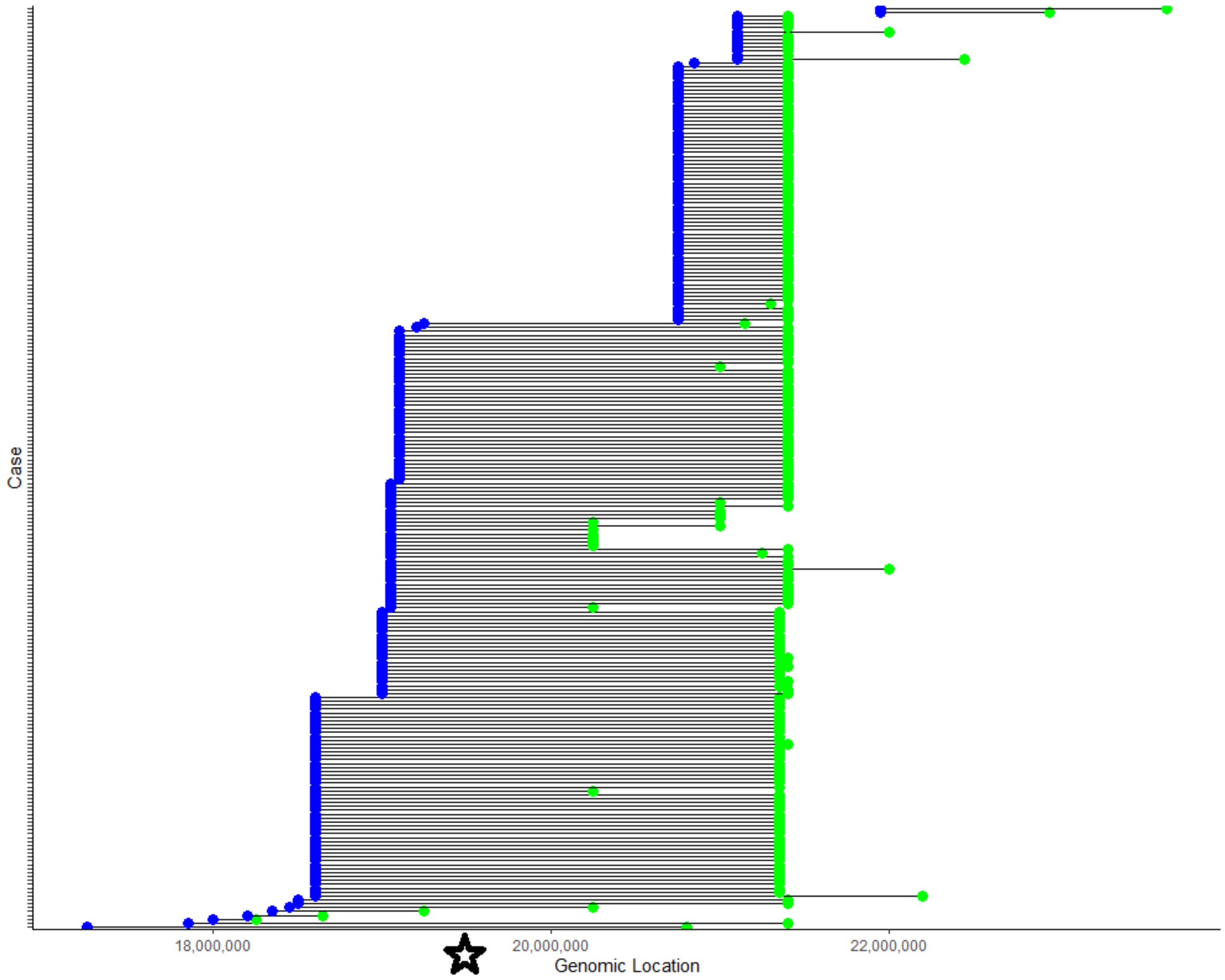

Supplemental Figure 2 – Three box and whisker plots showing the comparison of deletion size for

cases with and without ultrasound findings when (A) including all cases and (B) when removing suspected maternal cases and (C) looking at only confirmed fetal cases.

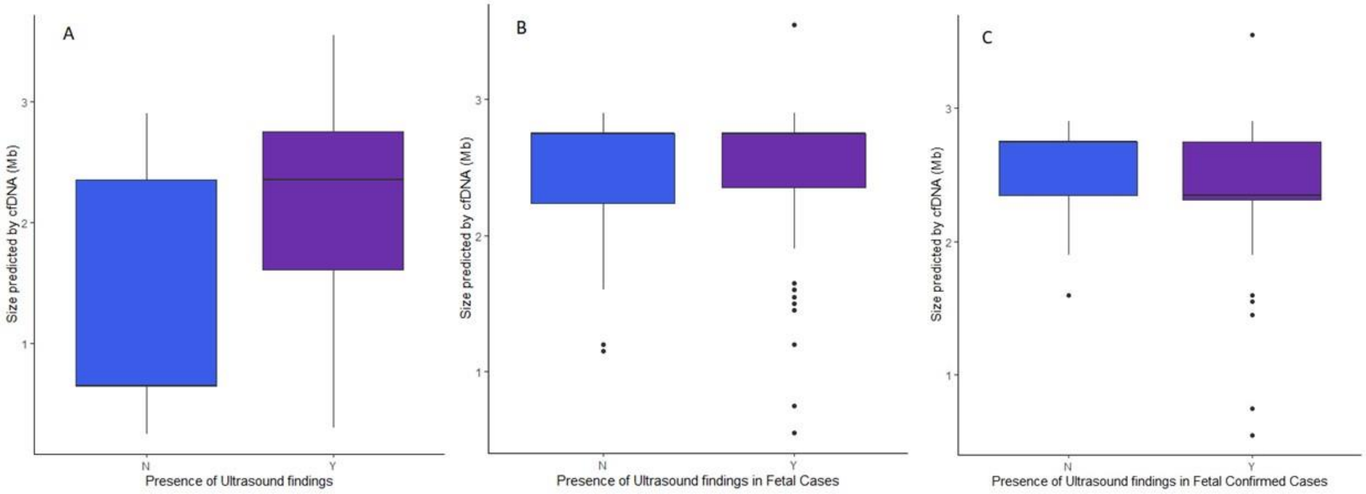

Supplemental Table 1 – Details of the 'incomplete' testing cases

	cfDNA details	Diagnostic testing details	Notes
1	RI: Ultrasound finding, testing at 21 weeks; Suspected **fetal** deletion; MR and cfDNA size NA	Maternal array and karyotype only (both negative); **no fetal testing**	Heart defect on ultrasound
2	RI: Ultrasound finding, testing at 25 weeks, Suspected **fetal** deletion; MR and cfDNA size NA	Parental arrays only (both negative); **no fetal testing**	TOF, polyhydramnios, and absent pulmonary valve on ultrasound
3	RI: Maternal age, testing at 21 weeks, Suspected **maternal** deletion; MR and cfDNA size NA	No fetal testing; maternal testing via FISH only (negative), **no microarray**	Atypical deletion predicted
4	RI: No known high-risk indication, testing at 16 weeks, Suspected **maternal** deletion; MR 13.448, 0.55 Mb deletion predicted	No fetal testing; maternal FISH and karyotype only (negative), **no microarray**	Provider noted *TUPLE1* probe was used; *HIRA* gene not included in region seen on cfDNA

5	RI: No known high-risk indication, testing at 11 weeks, Suspected **maternal** deletion; MR 14.21, 0.65 Mb deletion predicted	No fetal testing; maternal FISH only (negative), **no microarray**	Atypical deletion predicted
6	RI: Maternal age, testing at 12 weeks, Suspected **maternal** deletion; MR 10.162, 0.65 Mb deletion predicted	No fetal testing; maternal FISH only (negative), **no microarray**	Atypical deletion predicted
7	RI: No known high-risk indication, testing at 12 weeks, Suspected **maternal** deletion; MR 5.652, 0.65 Mb deletion predicted	No fetal testing; maternal blood karyotype only (negative), **no microarray**	Atypical deletion predicted
8	RI: Maternal age, testing at 10 weeks, Suspected **maternal** deletion; MR and cfDNA size NA	CVS array, karyotype, and FISH (negative), **no maternal testing**	Maternal hypothyroid disorder
9	RI: Maternal age, testing at 15 weeks, Suspected **maternal** deletion; MR 8.295, 2.35 Mb deletion predicted	Postnatal array (negative), **no maternal testing**	
10	RI: Maternal age, testing at 11 weeks, Suspected **maternal** deletion; MR 12.127, 0.65 Mb deletion predicted	CVS array (negative), **no maternal testing**	Atypical deletion predicted
11	RI: Ultrasound finding, testing at 24 weeks, Suspected **maternal** deletion; MR and cfDNA size NA	FISH/karyotype on amnio (both negative), no microarray and **no maternal testing**	2VC, CPC, Club foot on ultrasound
12	RI: Ultrasound finding, testing at 12 weeks, Suspected **maternal** deletion; MR 13.38, 2.35 Mb deletion	Karyotype on POC specimen after SAB, maternal balanced translocation confirmed (46,XX,t(16;18)(q13;p11.2)mat), no microarray and **no maternal microarray/FISH**	Ultrasound findings not specified; Maternal phenotype reported with severe LD/DD, dysmorphic facies reportedly

			consistent with 22q11.2DS
13	RI: Ultrasound finding, testing at 22 weeks, Suspected **maternal** deletion; MR and cfDNA size NA	FISH/karyotype on amnio (both negative), no microarray and **no maternal testing**	Bilateral CPCs
14	RI: Ultrasound finding, testing at 18 weeks, Suspected **maternal** deletion; MR: 8.29, cfDNA size NA	Amnio karyotype (negative), no microarray and **no maternal testing**	CPCs
15	RI: Ultrasound finding, testing at 30 weeks, Suspected **maternal** deletion; MR: 19.402, 0.65 Mb deletion	No fetal testing, Maternal FISH only (negative), **no microarray**	IUGR; Atypical deletion predicted
16	RI: None provided, testing at 10 weeks, Suspected **maternal** deletion; MR: 14.754, 0.65 Mb deletion	No fetal testing, Maternal karyotype only (negative), **no microarray**	Atypical deletion predicted
17	RI: No known high-risk indication, GA not provided, suspected **maternal** deletion; MR 15.49, 0.65 Mb deletion	FISH/karyotype on amnio (both negative), no microarray and **no maternal testing**	Atypical deletion predicted

cfDNA = cell-free DNA, RI = referral indication, MR = mosaicism ratio, Mb = megabase, NA = not available, TOF = tetralogy of fallot, FISH = fluorescent in situ hybridization, 2VC = two vessel cord, CPC = choroid plexus cyst(s), LD/DD = learning difficulties/developmental delay, 22q11.2DS = 22q11.2 deletion syndrome, IUGR = intrauterine growth restriction, GA = gestational age

Supplemental Table 2 – PPV calculations

	True positive	False Positive	PPV
Total cases with complete diagnostic testing (n=177)<br>• 75 suspected fetal cases with fetal testing (74 TP, 1 FP)<br>• 97 suspected maternal cases with maternal testing (97 TP)	176	1	99.4%<br>(95% CI: 96.4% - 99.9%)

• 5 suspected maternal cases with only fetal testing (5 TP)			
Treating 'incomplete' as FP (n = 194)	176	18	90.7%<br>(95% CI: 85.5% - 94.2%)
Cases with fetal testing + fetus suspected (minus maternal suspected cases) (n = 75)	74	1	98.7%<br>(95% CI: 91.8% - 99.9%)
Also including cases without diagnostic testing (n=307) as either:<br>• All FP<br>• All TP	176<br>306	131<br>1	57.3%<br>(95% CI: 51.6% - 62.9%)<br>99.7%<br>(95% CI: 97.9% - >99.9%)

PPV = positive predictive value, FP = false positive, TP = true positive

Supplemental Figure 3 – Flow chart summarizing the diagnostic testing for the 194 cases with diagnostic follow-up

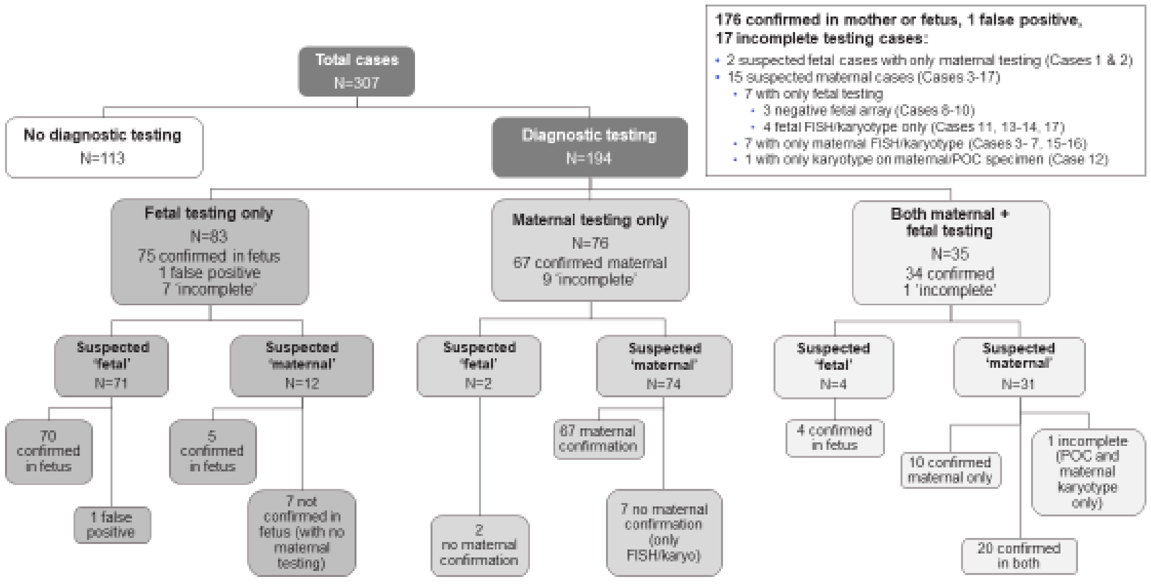

Supplemental Table 3 – Details of diagnostic testing specimens and assays.

No diagnostic testing	113
Confirmation in fetus/baby (specimen and assay type unspecified)	9
CVS FISH	4
CVS karyotype	2
CVS microarray	6
Amniotic fluid FISH	8
Amniotic fluid karyotype	13
Amniotic fluid microarray	35
Amniotic fluid - assay type unspecified	1
POC FISH	1
POC karyotype	2
POC microarray	3
Postnatal (Neonatal) FISH	12
Postnatal (Neonatal) microarray	31
Postnatal (Neonatal) - assay type unspecified	10
Maternal FISH	21
Maternal karyotype	6
Maternal microarray	58
Maternal testing - assay type unspecified	28

Note that most cases (n=137) had only one diagnostic test on a single specimen type, however, there were 48 cases that involved testing on either multiple assays or specimens (for example, both maternal and fetal microarray or both FISH and microarray on an amniotic fluid specimen), so the counts in the table are not mutually exclusive. CVS = chorionic villus sampling, FISH = fluorescence in situ hybridization, POC = products of conception

Supplemental Table 4 – Summary of the available details for cases with reported ultrasound findings

**Cardiac (Isolated)**		**71**
	TOF	22
	VSD	7
	Truncus arteriosus	4
	Aortic valve stenosis	1
	Complex or multiple cardiac anomaly	15

	Unspecified cardiac anomaly	20
**Cardiac + Renal**		**6**
	TOF + Pyelectasis	1
	Truncus arteriosus + Renal Anomalies NOS	1
	Right aortic arch with vascular ring + Multicystic dysplastic kidney	1
	Cardiac anomaly NOS + Pyelectasis	1
	Cardiac anomaly NOS + Renal anomalies NOS	1
	Cardiac anomaly NOS + Cystic kidney + Umbilical vein varix	1
**Cardiac + Additional findings (Non-Renal)**		**18**
	With facial cleft (Cardiac anomaly NOS x1)	1
	With fetal demise (Ductus arteriosus x1, Cardiac anomaly NOS x1)	2
	With polyhydramnios (TOF x2, Cardiac anomaly NOS x2)	4
	With IUGR (Truncus arteriosus x1, Cardiac anomaly NOS x1)	2
	With echogenic lungs (Cardiac anomaly NOS x1)	1
	With soft markers (Coarctation of the aorta/EIF x1, Aortic arch anomaly/EIF/Absent NB x1)	2
	Cardiac anomaly NOS + Shortened long bones + CNS anomaly NOS + Absent NB	1
	TOF + Absent thymus + Low set ears	1
	VSD + Polyhydramnios + Cleft palate	1
	Complex cardiac anomaly + Suspected esophageal atresia + Polyhydramnios	1
	Left-sided superior vena cava + 2VC + IUGR	1
	Complex cardiac anomaly + 2VC + Bilateral clubbed feet	1
**IUGR**		**11**
	Isolated IUGR	7
	With cleft lip	1
	With duodenal atresia	1
	With stillbirth	1
	With soft markers (EIF + 2VC)	1
**Fluid**		**4**
	Polyhydramnios	4
**Lymphatic**		**3**
	Increased NT	2

	Cystic hygroma + hydrops	1
**Nervous system**		
	Neural tube defect	2
**NOS**		**19**
**Other Structural Anomaly**		**3**
	Omphalocele	1
	Diaphragmatic hernia	2
**Renal**		**7**
	Pyelectasis	1
	Bilateral renal agenesis	1
	Anhydramnios + Renal anomalies NOS	1
	Oligohydramnios + Absent right kidney	1
	Unilateral renal agenesis + Unilateral club foot	1
	Bilateral hydronephrosis + Clubbed feet	1
	Multicystic kidney + Unilateral urinary tract dilatation + Bilateral CPCs	1
**Skeletal**		**2**
	Unilateral clubfoot	1
	Bilateral clubbed feet + Polyhydramnios	1
**Soft markers**		**7**
	CPCs	2
	Absent NB	1
	EIF + 2VC	1
	CPC + EIF	1
	EIF	1
	EIF + Echogenic bowel + Fetal demise	1
**Multisystem (non-cardiac)**		**6**
	2VC + CPC + Clubfoot	1
	MCA NOS	3
	Polyhydramnnios + Abnormal ear + Clinodactyly	1
	Polyhydramnios + Unilateral clubbed foot + Sandal-gap toe + Unable to see stomach	1

TOF = tetralogy of fallot, VSD = ventricular septal defect, NOS = Not otherwise specified, IUGR = intrauterine growth restriction, EIF = echogenic intracardiac focus, NB = nasal bone, CNS = central nervous system, 2VC = two vessel cord, NT = nuchal translucency, CPCs = choroid plexus cysts, MCA = multiple congenital anomalies

Supplemental Table 5 – Details of the cases with additional findings from microarray

Case	Assay details	Additional array finding(s)
Case S1	Maternal blood array	1.61 Mb interstitial duplication of 9q33.1; 570 kb interstitial duplication of 2p16.3
Case S2	POC + cord blood array	1.33 Mb interstitial duplication of 17p12
Case S3	Maternal blood array	853 kb interstitial duplication of Xp22.2
Case S4	Maternal blood array	700 kb gain of 4p16.1
Case S5	Maternal blood array	853 kb interstitial duplication of Xp22.2
Case S6	Amniotic fluid array	546 kb interstitial duplication of 15q26.3
Case S7	Maternal blood array	855 kb interstitial deletion of 15q11.2
Case S8	Maternal blood array	472 kb interstitial duplication of 12q23.2
Case S9	Neonatal array	255 kb interstitial deletion of 20p12.1
Case S10	Maternal blood array	667 kb interstitial duplication of 6q27
Case S11	Neonatal array	255 kb interstitial deletion of 6p25.1
Case S12	Maternal blood array	94 kb interstitial deletion of 9p24.1
Case S13	Maternal blood array	"Small duplication on 15q" – verbal report from provider
Case S14	Maternal blood array	2.09 Mb interstitial duplication of 20q13.33
Case S15	Maternal blood array	667 kb interstitial duplication of 6q27

Case S16	Maternal blood array	22 kb interstitial deletion of 14q31.3

POC = products of conception, Mb = Megabases, kb = kilobases

Supplemental Table 6: Clinical details of cases without diagnostic testing.

**Case Identifier**	**Screening indication**	**Suspected origin on cfDNA sequencing data**	**Ultrasound findings reported?**	**If yes, details:**	**Notes**
6	Maternal age, Ultrasound finding	Fetal	Y	NOS	
11	Ultrasound finding	Fetal	Y	TOF, pyelectasis	Patient moved, no follow-up
18	Personal or family history	Fetal	N		
25	Ultrasound finding	Fetal	Y	IUGR	
33	Abnormal serum biochemical screening, Ultrasound finding	Maternal	Y	Truncus arteriosus and renal anomalies	
35	Ultrasound finding	Fetal	Y	IUGR, cleft lip	
37	Maternal age	Maternal	N		Declined testing

38	Ultrasound finding	Fetal	Y	NOS	LTFU
40	Ultrasound finding	Maternal	Y	Anhydramnios and renal anomalies	TOP, no POC testing
41	No known high risk	Maternal	N		LTFU
43	Ultrasound finding	Fetal	Y	TOF	
44	Maternal age	Maternal	N		
47	Maternal age, Abnormal serum biochemical screening	Maternal	N		
57	Maternal age	Maternal	N		
58	Maternal age	Maternal	N		
59	Maternal age	Maternal	N		TOP, no testing
60	Maternal age	Maternal	N		
62	Ultrasound finding	Fetal	Y	Polyhydramnios	
64	Abnormal serum biochemical screening	Maternal	N		

68	None provided	Maternal	N		
72	Maternal age	Maternal	N		
75	Maternal age	Maternal	N		
78	Ultrasound finding	Fetal	Y	Polyhydramnios, cleft palate, VSD	
79	Ultrasound finding	Fetal	Y	Complex heart defect	
82	Maternal age	Fetal	N		
86	Ultrasound finding, Maternal age	Maternal	Y	Heart defect NOS	
91	Abnormal serum biochemical screening, Ultrasound finding	Maternal	Y	NOS	
93	Maternal age	Maternal	N		
94	Ultrasound finding	Maternal	Y	Multiple congenital anomalies	cfDNA also positive for trisomy 21
95	Maternal age	Maternal	N		
97	Ultrasound finding	Maternal	Y	NOS	

98	Ultrasound finding	Fetal	Y	NOS	
105	Ultrasound finding	Maternal	Y	IUGR	
111	Maternal age	Maternal	N		
112	None provided	Maternal	N		
116	Ultrasound finding	Fetal	Y	VSD	
120	Personal or family history	Maternal	N		
125	Maternal age	Maternal	N		
127	Ultrasound finding	Fetal	Y	Truncus arteriosus, VSD	
128	Ultrasound finding	Fetal	Y	Fetal cardiac anomalies NOS, pulmonary stenosis / VSD/ ? aortic stenosis	
135	Ultrasound finding	Fetal	Y	EIF and 2VC	
137	Maternal age	Fetal	N		
139	Ultrasound finding	Fetal	Y	2VC, left SVC, SGA	

140	Ultrasound finding	Fetal	Y	TOF	
142	Ultrasound finding	Fetal	Y	Heart defect NOS and polyhydramnios	
144	Ultrasound finding	Fetal	Y	Truncus arteriosus	
145	Ultrasound finding	Fetal	Y	Heart defect NOS	
146	Ultrasound finding	Fetal	Y	Heart defect NOS and renal anomalies	
148	Maternal age	Maternal	N		Egg donor gestation so fetus not at risk
151	Ultrasound finding	Fetal	Y	Heart defect NOS, IUFD	
152	Maternal age	Maternal	N		
155	Maternal age	Maternal	N		
158	None provided	Fetal	N		
160	Maternal age	Maternal	N		
164	Ultrasound finding	Fetal	Y	TOF	
167	Maternal age	Maternal	N		

168	Maternal age	Maternal	N		
170	Ultrasound finding	Maternal	Y	CPC, EIF	
172	No known high risk	Maternal	N		
174	Maternal age	Fetal	N		
177	Maternal age	Maternal	N		
178	Maternal age	Fetal	N		
179	Ultrasound finding	Fetal	Y	Heart defect NOS	
180	Ultrasound finding	Fetal	Y	Heart defect NOS	
181	Maternal age	Fetal	N		
182	Ultrasound finding	Fetal	Y	NOS	
184	No known high risk	Fetal	Y	Right sided aortic arch with vascular ring	
187	Ultrasound finding	Fetal	Y	VSD; small aortic valve and arch	Negative maternal karyotype
188	Ultrasound finding	Fetal	Y	TOF	

190	Maternal age	Maternal	N		Prior child with DiGeorge (unclear whether diagnosis based on clinical features or confirmed with testing)
191	Ultrasound finding	Fetal	Y	TOF	LTFU
194	Maternal age	Maternal	N		
198	Abnormal serum biochemical screening, Personal or family history	Maternal	N		Prior child with DiGeorge (unclear whether diagnosis based on clinical features or confirmed with testing)
199	Ultrasound finding	Fetal	Y	Truncus arteriosus, IUGR	
200	Ultrasound finding	Fetal	Y	TOF	
203	Ultrasound finding	Fetal	Y	Heart defect NOS	
204	Ultrasound finding	Fetal	Y	Unilateral club foot; sandle-gap toe; polyhydramnios; didn't visualize stomach.	
206	Ultrasound finding	Fetal	Y	Complex cardiac defect	

211	Ultrasound finding	Maternal	Y	Heart defect NOS; echogenic lungs	
212	Ultrasound finding	Maternal	Y	EIF left ventricle	
217	None provided	Maternal	N		
219	No known high risk	Maternal	N		
222	No known high risk	Maternal	N		Patient tested with subsequent pregnancy with similar findings
223	Personal or family history	Maternal	N		
229	Ultrasound finding	Maternal	Y	Bilateral renal agenesis	
231	Ultrasound finding	Fetal	Y	TOF	
233	Maternal age	Maternal	N		
236	Ultrasound finding	Maternal	Y	NOS	Patient had TOP; Severe diabetic
239	Ultrasound finding, Abnormal serum biochemical screening	Fetal	Y	TOF, absent pulmonary valves	

240	Ultrasound finding	Fetal	Y	EIF, absent nasal bone, aortic arch	
243	Ultrasound finding	Fetal	Y	TOF and major aortopulmonary collateral anastomoses	
252	Ultrasound finding	Fetal	Y	VSD and aortic stenosis	
257	Personal or family history	Maternal	N		
258	Ultrasound finding	Fetal	Y	NOS	
263	Maternal age	Maternal	N		
265	No known high risk	Fetal	N		
266	Maternal age, Ultrasound finding	Fetal	Y	Heart defect NOS	
270	Ultrasound finding	Maternal	Y	Diaphragmatic hernia	
274	Maternal age	Fetal	N		
277	No known high risk	Maternal	N		

282	Ultrasound finding	Fetal	Y	Heart defect NOS	
283	None provided	Maternal	N		Declined testing
285	No known high risk	Maternal	N		
286	None provided	Maternal	N		
289	Ultrasound finding	Maternal	Y	NOS	
291	Ultrasound finding	Fetal	Y	NOS	
295	No known high risk	Maternal	N		Normal ultrasound at 15 weeks; patient declined testing
297	No known high risk	Maternal	N		
299	None provided	Maternal	N		
301	None provided	Maternal	N		Patient declined testing, normal anatomy scan
304	No known high risk	Maternal	N		
305	Ultrasound finding	Fetal	Y	NOS	

307	No known high risk	Maternal	N		

TOF = tetralogy of fallot, VSD = ventricular septal defect, NOS = Not otherwise specified, IUGR = intrauterine growth restriction, EIF = echogenic intracardiac focus, NB = nasal bone, CNS = central nervous system, 2VC = two vessel cord, NT = nuchal translucency, CPCs = choroid plexus cysts, LFTU = lost to follow-up, TOP = termination of pregnancy, SGA = small for gestational age, SVC = superior vena cava, IUFD = intrauterine fetal demise

Supplemental Table 7: Ultrasound details for suspected fetal cases with diagnostic testing (74 true positive fetal cases and 1 false positive case)

**Case Identifier**	**Ultrasound findings reported?**	**If yes, details:**	**Classification**
1	Y	TOF	True Positive
2	Y	TOF	True Positive
5	Y	Heart defect (unspecified)	True Positive
10	Y	Heart defect (unspecified), shortened long bones, CNS abnormality (unspecified), absent nasal bone	True Positive
13	Y	TOF	True Positive
21	Y	Thick NT	True Positive
22	Y	Myelomeningocele	True Positive
26	Y	VSD, coarctation of the aorta	True Positive
30	Y	NOS	True Positive
34	Y	NOS	True Positive

36	Y	Heart defect (unspecified)	True Positive
39	Y	VSD	True Positive
42	Y	IUGR	True Positive
49	N		True Positive
51	Y	Heart defect (unspecified)	True Positive
61	Y	Polyhydramnios, heart defect (unspecified)	True Positive
65	Y	VSD	True Positive
66	Y	Pyelectasis	True Positive
67	N		True Positive
70	N		True Positive
74	Y	IUGR, duodenal atresia	True Positive
80	Y	Heart defect (unspecified)	True Positive
81	Y	TOF	True Positive
83	Y	Heart defect (unspecified)	True Positive
87	Y	Truncus arteriosus	True Positive
89	Y	Polyhydramnios	True Positive
90	Y	TOF	True Positive

100	Y	Heart defect (unspecified)	True Positive
101	Y	Cystic hygroma, hydrops	True Positive
102	Y	Coarctation of aorta and EIF	True Positive
104	Y	Right aortic arch, vascular ring, multicystic dysplastic kidney	True Positive
107	Y	VSD	True Positive
110	N		True Positive
113	Y	Unilateral renal agenesis, unilateral club foot	True Positive
115	Y	Echogenic bowel, EIF, IUFD	True Positive
117	Y	NOS	True Positive
118	Y	Ductus arteriosus; infant demise	True Positive
126	Y	Heart defect and pyelectasis	True Positive
129	Y	TOF	True Positive
130	Y	2VC, complex heart defect (unspecified), polyhydramnios, esophageal atresia?, perinatal demise	True Positive
131	Y	Heart defect (unspecified)	True Positive
132	N		True Positive
133	N		True Positive

136	Y	Heart defect (unspecified); IUGR	True Positive
138	N		True Positive
141	Y	Unilateral clubfoot	True Positive
147	N		True Positive
154	Y	Omphalocele	True Positive
156	Y	Polyhydramnios, abnormal ear, clinodactyly	True Positive
161	Y	TOF, polyhydramnios	True Positive
165	Y	Bilateral clubbed feet, 2VC, VSD, possible transposition of great arteries	True Positive
169	Y	Heart defect (unspecified)	True Positive
171	N		False Positive
173	N		True Positive
175	N		True Positive
176	Y	TOF	True Positive
186	N		True Positive
202	Y	Cystic kidney; umbilical vein varix; possible heart defect (unspecified)	True Positive
208	Y	Complex heart defect (unspecified)	True Positive

218	Y	Complex heart defect (unspecified)	True Positive
220	Y	TOF	True Positive
224	Y	TOF	True Positive
232	Y	TOF	True Positive
237	Y	TOF with right aortic arch	True Positive
238	Y	Heart defect (unspecified)	True Positive
247	Y	TOF	True Positive
249	Y	Heart defect (unspecified), cleft lip	True Positive
254	Y	IUGR, 2VC, EIF	True Positive
261	N		True Positive
262	Y	Severe polyhydramnios	True Positive
267	Y	Bilateral clubbed feet and polyhydramnios	True Positive
269	Y	Heart defect (unspecified)	True Positive
276	Y	VSD, vascular ring and aberrant right subclavian artery	True Positive
278	Y	TOF	True Positive
292	Y	VSD	True Positive

TOF = tetralogy of fallot, VSD = ventricular septal defect, NOS = Not otherwise specified, IUGR = intrauterine growth restriction, EIF = echogenic intracardiac focus, NB = nasal bone, CNS = central nervous system, 2VC = two vessel cord, NT = nuchal translucency
